# Fabrication and Characterization of Nanoenergetic Hollow Spherical Hexanitrostibene (HNS) Derivatives

**DOI:** 10.3390/nano8050336

**Published:** 2018-05-16

**Authors:** Xiong Cao, Peng Deng, Shuangqi Hu, Lijun Ren, Xiaoxia Li, Peng Xiao, Yu Liu

**Affiliations:** 1 School of Environment and Safety Engineering, North University of China, Taiyuan 030051, China; nash_deng@163.com (P.D.); hsq@nuc.edu.cn (S.H.); rlj0907@163.com (L.R.); lxxjy0506@163.com (X.L.); itimeless2018@163.com (P.X.); 2 Institute of Chemical Materials, China Academy of Engineering Physics, Mianyang 621900, China

**Keywords:** HNS, spherical nanoparticles, derivatives, hollow, sensitivity

## Abstract

The spherization of nanoenergetic materials is the best way to improve the sensitivity and increase loading densities and detonation properties for weapons and ammunition, but the preparation of spherical nanoenergetic materials with high regularization, uniform size and monodispersity is still a challenge. In this paper, nanoenergetic hollow spherical hexanitrostibene (HNS) derivatives were fabricated via a one-pot copolymerization strategy, which is based on the reaction of HNS and piperazine in acetonitrile solution. Characterization results indicated the as-prepared reaction nanoenergetic products were HNS-derived oligomers, where a free radical copolymerization reaction process was inferred. The hollow sphere structure of the HNS derivatives was characterized by scanning electron microscopy (SEM), transmission electron microscope (TEM), and synchrotron radiation X-ray imaging technology. The properties of the nanoenergetic hollow spherical derivatives, including thermal decomposition and sensitivity are discussed in detail. Sensitivity studies showed that the nanoenergetic derivatives exhibited lower impact, friction and spark sensitivity than raw HNS. Thermogravimetric-differential scanning calorimeter (TG-DSC) results showed that continuous exothermic decomposition occurred in the whole temperature range, which indicated that nanoenergetic derivatives have a unique role in thermal applications. Therefore, nanoenergetic hollow spherical HNS derivatives could provide a new way to modify the properties of certain energetic compounds and fabricate spherical nanomaterials to improve the charge configuration.

## 1. Introduction

With the rise in international conflicts, modern weapons require more from energetic materials [1,2], such as higher energy, insensitivity, effective destructibility, and so on. To meet these needs, many strategies have been employed in the field of energetic materials [3,4,5,6,7,8], mainly including the synthesis of new explosives, and modification of existing energetic materials. Synthesis of new explosives involves complicated processing conditions, is time consuming and requires a comprehensive consideration of cost and suitability for final military applications, in which some problems still exist to a certain extent [9,10]. On the other hand, modification of existing energetic materials is an efficient method to enhance explosive performance, or desensitize existing explosives and improve safety. Despite its impact on energy and the environment, which is slight compared to the synthesis of new explosives [11,12], this strategy provides a direct way to realize expected goals [13]. Among feasible modification technologies, the spherization technology of nanoenergetic materials, has become a popular research topic and the design and fabrication of energetic materials has attracted considerable attention from both academic and industrial fields [14,15,16].

Spherization nanotechnology has always been considered as a significant method to enhance micro morphology, control micro structure and transform the properties of materials for potential applications [17,18,19,20]. Especially in the field of energetic materials, the spherization of nanoenergetic materials, which is of great benefit to improve free-flowing properties of the material, to optimize particle gradation, avoid cracking, further increase the charge density and even the detonation energy, has been widely studied and developed [14,21,22,23,24,25]. For instance, Vijayalakshmi [26] reported the use of a cooling crystallization technique to fabricate spherical 3-nitro-1,2,4-triazol-5-one (NTO) particles in a ternary system, where a selective particle size distribution ranging from 10 to 200 μm could generally be achieved. Bayat [27] introduced sonication into spaying-assisted solvent/nonsolvent crystallization to prepare spherical or ellipsoidal hexanitrohexaazaisowurtzitane (CL-20) nanoparticles with an average size of 95 nm, whose impact sensitivity was decreased in comparison to a micrometer-size CL-20. More interestingly, Huang [28] demonstrated the use of electrospray deposition nanotechnology to prepare 2,6-diamino-3,5-dinitropyrazine-1-oxide (LLM-105) sub-microspheres with diameters ranging from 200–500 nm composed of 50 nm nanoparticles, which have a lower onset degradation temperature compared to raw materials. However, it can be found from these studies that although as-reported nanoparticles possess a certain degree of sphericity, they are closer to ellipsoid shapes with a wide size distribution and they can be badly agglomerated [29,30,31] because of incomplete control technology and the inherent nature of small organic molecule materials. So, the development of spherical nanoenergetic materials remains an enormous challenge [32]. There is an urgent need to develop a new spherization nanotechnology to solve these problems.

In previous works, our group concentrated on design and modification of energetic material HNS at molecule level and at micro scale [33,34]. A surprising phenomenon, self-assembly of hollow spherical HNS derivatives, was found unexpectedly and various synthesis parameters have been systematically investigated to obtain the optimized conditions [35]. Although it is meaningful to fabricate spherical nanoenergetic materials, there are still many related scientific problems, which need to be solved further. In this paper, we demonstrate a one-pot copolymerization strategy to fabricate nanoenergetic hollow spherical HNS derivatives. To the best of our knowledge, this distinctive method with its simple operational technique not only avoids complex condition control but achieves precise and efficient control of nanomaterial microstructures, and we produced as-prepared nanoenergetic derivatives, which possessed highly regularized, uniform size distribution and good monodispersity. This was reported first, then compared to other reports mentioned above [15,26] and then the properties of the nanoenergetic derivatives were studied systemically for future applications. This work introduces a new concept into the field of spherization nanotechnology for the design and fabrication of other spherical nanomaterials.

## 2. Experimental

### 2.1. Materials

**Figure 1:**
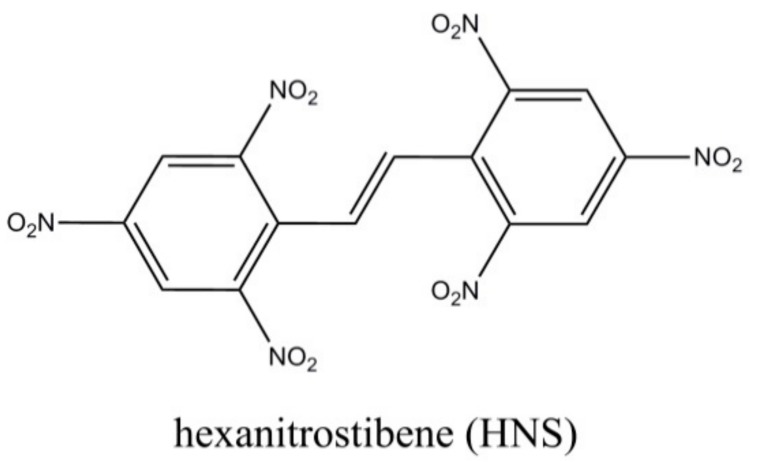
The chemical structure of the hollow spherical hexanitrostibene (HNS).

HNS-II (as chemical structure shown in Figure 1) was provided from China Academy of Engineering Physics. Piperazine was purchased from Shanghai Aladdin Co., Ltd., Shanghai, China. Acetonitrile was purchased from Chengdu Kelong Chem. Corp., Chengdu, China. All chemicals were used as received without any further purification. Deionized water was used in all experiments.

### 2.2. Sample Preparation

In a typical experiment, 0.450 g HNS, 0.387 g piperazine (molar ratio: 1:4.5) and 180 mL acetonitrile were added to a flask together. The mixture was moved into a water bath at a temperature of 80 °C with continuous stirring at 150 rpm and allowed to react completely for 5 h. The solution was naturally cooled down to room temperature and then the sample was obtained through filtration.

### 2.3. Characterization

Scanning electron microscopy (SEM) images were collected by the Sigma microscope. Samples were dispersed in deionized water, drop-cast onto a silicon chip and dried before analysis. Transmission electron microscope (TEM) images were detected in a Libra 200 microscope. Samples were dispersed in deionized water, drop-cast onto a carbon-coated microgrid and dried naturally before analysis. Synchrotron radiation X-ray imaging technology images were obtained by the X-ray scattering apparatus (National Synchrotron Radiation Light, Hefei, China). XRD patterns were recorded on a Bruker D8 Advance X-ray diffractometer using Cu Kα radiation (40 kV, 40 mA). Fourier transform infrared (FTIR) spectra were collected on a Nicolet 6700 Fourier spectrometer, using KBr pellets of the solid samples. Nuclear Magnetic Resonance (NMR) data were obtained by Bruker Avance 600, using CD_3_CN as a deuterium reagent.

Thermal analysis was carried out on a TG-DSC calorimeter (Netzsch STA449F3, Germany) at heating rates of 10 °C min^−1^. The impact sensitivity was tested with a WL-1 type impact sensitivity apparatus. The special height (H_50_) represents the height from which a 2.000 ± 0.002 kg drop-hammer will result in an explosive event in 50% of the trials. The test conditions were: sample mass of 30 mg; 25 drop tests were completed to calculate the H_50_. The friction sensitivity experiments were measured with a WM-1 type friction sensitivity instrument. The test conditions were: relative pressure of 4.92 MPa; sample mass of 30 mg; pendulum weight of1.5 kg; pendulum angle of 90°. Twenty-five tests were completed to give the probability of explosion (%). The electrostatic spark sensitivity experiments were tested by a JGY-50 type electrostatic sensitivity apparatus.

## 3. Results and Discussion

**Figure 2:**
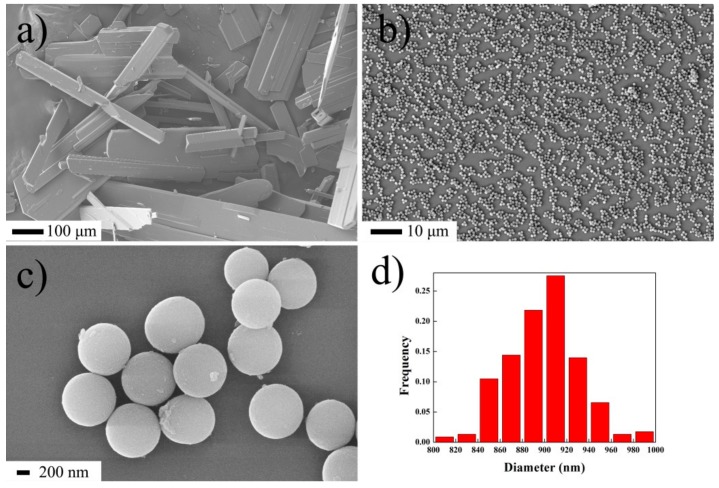
The SEM images of (**a**) raw HNS and (**b**,**c**) as-prepared samples, (**d**) the particle size distribution of spherical nanoparticles from statistical data by SEM.

The morphologies of raw HNS and as-prepared nanoenergetic derivative samples based on the optimized conditions [35] are shown in Figure 2. Raw HNS exhibited bulk shapes with an irregular size ranging from micro-scale to millimeter-scale (Figure 2a). However, piperazine cannot be observed because of the hygroscopicity of CO_2_ and H_2_O. The samples shown in Figure 2b,c are produced by the reaction of HNS and piperazine in acetonitrile solution. From Figure 2b, it can be seen that the nanoparticles replaced the bulk materials. These nanoparticles were dispersed randomly without aggregation on the surface of the silicon chip, which indicates the good monodispersity of nanoparticles. By the use of local magnification in Figure 2c, it can be seen that almost all the particles have a highly regular spherical shape. The particle size distribution was counted by collecting the diameter of 200 targets from SEM data, as showed in Figure 2d. The average diameter of nanoparticles ranged from 800 nm to 1000 nm with an average diameter of about 900 nm. Also, most of the nanoparticles were highly uniform spheres with a relative standard deviation below 5%, that is, the nanosphere diameter ranged from 850 nm to 950 nm. To the best of our knowledge, despite recent progress in the synthesis of uniform nanospheres in the materials field, micro- or nanospheres in energetic materials usually show a serious nanoparticle aggregation, a relatively broad (above 5%) size distribution or yield irregular, non-spherical shapes. This current work is significant because it reports demonstrated highly regular spherical nanoenergetic materials with high monodispersion and a narrow particle size distribution with a relative standard deviation below 5%.

**Figure 3:**
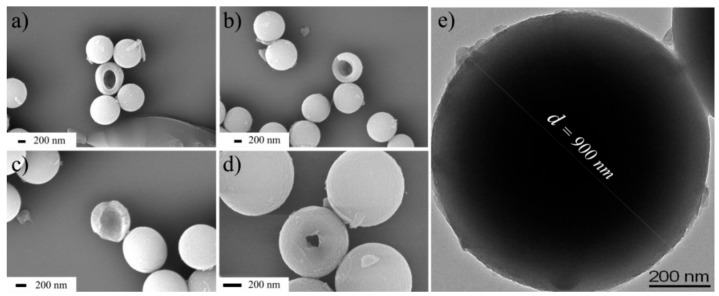
(**a**–**d**) the SEM images and (**e**) the TEM image of the spherical nanoenergetic derivatives.

In order to directly describe the inner structure of nanoenergetic spherical derivatives, a small amount of broken spherical nanoparticles which were ground slightly in the deionized water can be observed in Figure 3a–d. The broken nanoparticles which were squeezed and sheared randomly, provide clear evidence of the inner hollow structure and the shell wall thickness of the damaged spherical nanoparticles, which is up to 100 nm (Figure 3c). Then, a spherical nanoparticle with a diameter of 900 nm was characterized by TEM (shown in Figure 3e). Some differences existed in the contrast ratio when comparing the surrounding margins with the middle parts of the spherical nanoparticles. However, due to their thicker shells (above 100 nm), the TEM image of spherical nanoparticles cannot provide direct evidence to effectively illustrate their inner structure.

**Figure 4:**
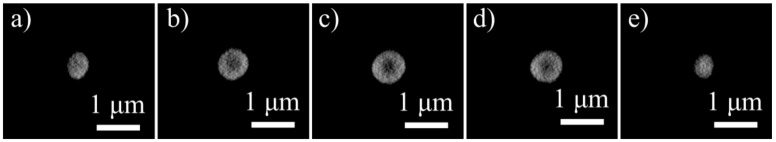
(**a**–**e**) A group of synchrotron radiation X-ray imaging technology images of spherical nanoenergetic derivatives.

Furthermore, synchrotron radiation X-ray imaging technology analysis was utilized to investigate the inner structure of spherical nanoparticles. A group of synchrotron radiation X-ray imaging technology images of spherical nanoenergetic derivatives are collected in Figure 4 to demonstrate the inner structure of the nanoenergetic spherical nanoparticles by randomly testing single samples. Synchrotron radiation X-ray imaging technology is a new nano-CT technology with higher resolution. By scanning the different regions, a series of tomographical information can be obtained as shown by the group of images in Figure 4. With this information, it was possible to compare the intermediate section (Figure 4c) with the beginning or end section (Figure 4a or Figure 4e, respectively) and verify the differences in the inner hollow structures of nanoparticles.

**Figure 5:**
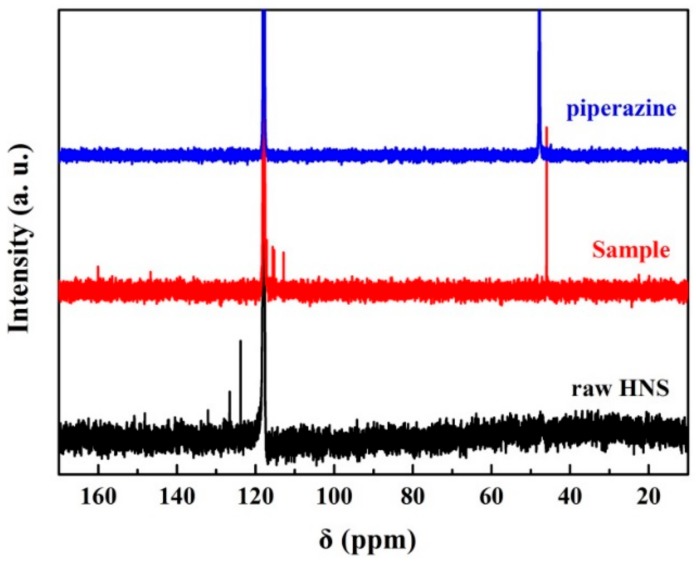
The ^13^C NMR of raw HNS (black), piperazine (blue) and the sample: nanoenergetic derivatives (red), using acetonitrile-d_2_ as a deuterium reagent.

In the ^13^C NMR patterns shown in Figure 5, raw HNS, piperazine and nanoenergetic derivatives were investigated. Considering that many reaction products have poor solution ability in reagents, acetonitrile-d_2_ with relatively good solubility was chosen as a deuterium reagent for NMR tests. From the results, the ^13^C NMR spectrum of nanoenergetic derivatives showed resonance peaks from aromatic carbons ranging from 170 ppm to 110 ppm and piperazine carbon at 45.7 ppm, suggesting that the products were synthesized from the reaction of HNS and piperazine. Compared to HNS, the resonance peaks of the nanoenergetic derivatives have shifted although the benzene skeleton structure remains stable. For piperazine segments of nanoenergetic derivatives, four piperazine carbon atoms have only one resonance peak shift from raw piperazine (the resonance peak at 48 ppm), indicating that these carbon atoms are in the same chemical environment. Many complex chemical reactions for nitro-aromatic compounds are involved in alkaline environments: HNS is a typical nitro-aromatic compound and piperazine is an alkaline substance [36]. Under this reaction condition, the piperazine radicals are formed easily because of the properties of organic alkaline substances. As alkaline active sites, piperazine radicals attacked C=C bonds, which are regarded as weak points in the chemical structure of HNS. More interestingly, the piperazine radical with two active sites can induce the main cross-linking chain reactions with C=C bonds via free radical copolymerization reaction, indicating that HNS-piperazine derived oligomers may be formed in the end.

Based on the above results, the formation mechanism of nanoenergetic hollow spherical HNS derivatives can be proposed: by simple one-pot copolymerization reactions, piperazine free radical reacted with HNS firstly, and HNS derivatives were continuously formed Partial electric charges at the surfaces accumulated and HNS derivatives occurred by agglomeration. Due to mutual repulsion of surface charges, agglomerated derivatives bent gradually, rolled up and ultimately formed primary hollow structures. Then, primary hollow nanospheres continued to grow as more derivatives were adsorbed until the surface charge stabilized. Finally, the as-obtained nanoenergetic hollow spherical HNS derivatives maintained high colloid stability.

**Figure 6:**
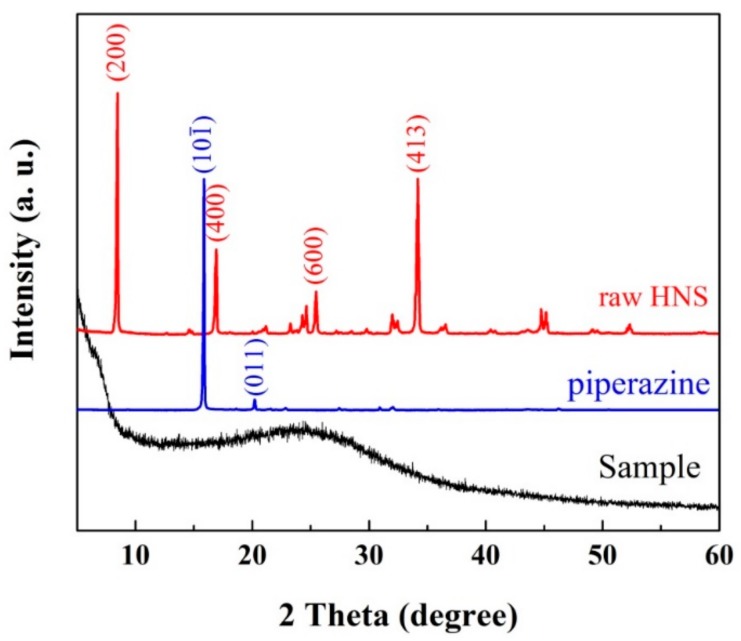
The XRD patterns of raw HNS (red), piperazine (blue) and the sample: nanoenergetic derivatives (black).

Figure 6 shows the XRD patterns of as-prepared samples. For raw materials, the red pattern demonstrated that the main diffraction peaks at the degrees of 8.4, 16.9, 25.4, and 34.1 corresponded to the (200), (400), (600) and (413) plane, respectively, of the HNS crystal, and also overlapped with data from JCPDS card No. 00-042-1919. Also, the XRD pattern of piperazine showed that the two main diffraction peaks at the degrees of 15.9 and 20.2 corresponded to the (10$\bar{1}$) and (011) plane of the piperazine crystal. However, compared with highly-crystalline raw materials, the low-level crystalline demonstrated the nanoenergetic derivatives attributes of amorphous material, which could indirectly prove that the reaction products were HNS-derived oligomers.

**Figure 7:**
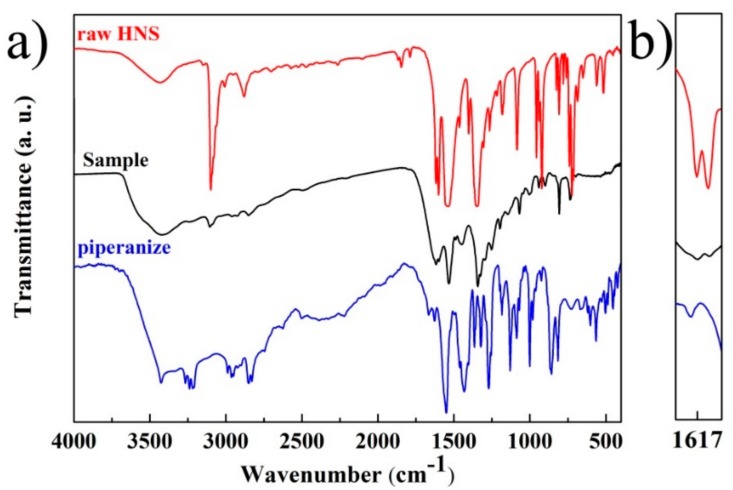
(**a**) The FT-IR patterns of raw HNS (red), piperazine (blue) and the sample: nanoenergetic derivatives (black), (**b**) Magnified FT-IR patterns from (**a**).

Furthermore, the as-prepared samples were studied by FT-IR, as shown in Figure 7. The distinct peaks of raw HNS are as follows [34]: 3098 cm^−1^ (aromatic C–H stretching), 2880 cm^−1^ (alkenic C–H str), 1618 cm^−1^ (aromatic C=C str), 1600 cm^−1^ (alkenic C=C str), 1540 cm^−1^ (NO_2_ asymmetric str), 1345 cm^−1^ (NO_2_ symmetric str), and so on. The distinct peaks of piperazine are as follows: 922 cm^−1^ (ring skeleton str), 1430 cm^−1^ (–CH_2_– bending vibrations), and so on. For the FT-IR spectrum of reaction products, the absorption peaks at 1617 cm^−1^, 1599 cm^−1^, 1532 cm^−1^ and 1340 cm^−1^, as well as 1448 cm^−1^, 940 cm^−1^ demonstrated that the main functional groups, such as the aromatic ring skeleton, nitro groups and piperanize ring skeleton and its –CH_2_– unit, have remained. Compared with raw HNS (shown in Figure 7b), the relative intensity of absorption peaks between aromatic C=C bonds (1617 cm^−1^) and alkenic C=C bonds (1599 cm^−1^) underwent a remarkable change and this indicated that raw HNS participated in the reaction with piperazine radicals mainly through C=C bonds [37,38]. This is in accordance with the ^13^C NMR results. However, the intensity of alkenic C=C bonds (1599 cm^−1^) absorption decreased but remained, which suggested that side reaction pathways for HNS also existed in the mixed system.

**Figure 8:**
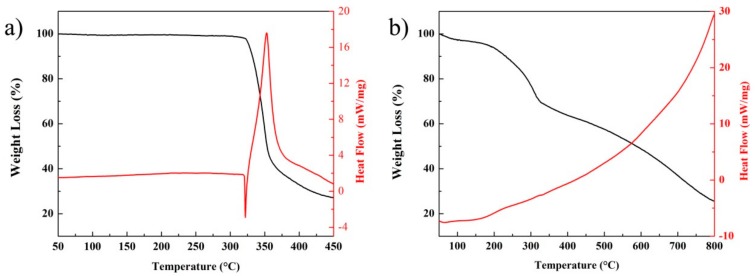
TG-DSC curves of the samples: (**a**) raw HNS and (**b**) nanoenergetic HNS derivatives in N_2_ air flow and a heat rate of 10 °C min^−1^.

The thermal performances of the samples were investigated by thermogravimetric-differential scanning calorimetry (TG-DSC) measurement, as shown in Figure 8. For the pure HNS crystal in Figure 8a, the high thermal stability of the pure HNS (the initial thermal decomposition temperature is 332 °C) is shown, which originates from the rich intramolecular and intermolecular interactions, especially the hydrogen bonding. However, nanoenergetic HNS derivatives exhibited attractive and interesting thermal properties based on their thermal decomposition. Compared to the thermostable explosive HNS, a continuous thermal decomposition process was discovered, shown in Figure 8b, which was in agreement with thermal decomposition of polymer or oligomer materials [39,40]. Slow thermal decomposition occurred in the full range from 50 °C to 800 °C with the release of heat. During this process, the mass loss of sample is about 80%. Although the thermal stability of nanoenergetic derived material is relatively unsatisfactory, its unique decomposition action and slow energy release process may provide new application prospects in particular environments.

In order to analyze the safety of the sample, impact, spark, and friction sensitivity tests were performed, and the results are listed in Table 1. For impact sensitivities as listed in Table 1, the special height (H_50_) of nanoenergetic hollow derivatives is more than 112.2 cm, which exhibited insensitivity property, compared to the raw HNS (39.5 cm) and even other explosives, such as CL-20 (29.4 cm), cyclotetramethylenete tranitramine (HMX, 63.0 cm) and cyclotrimethylene trinitramine (RDX, 99.1 cm), where the samples were tested using a 2 kg drop-hammer, and a 30 mg sample mass. This is because spherical nanoenergetic derivatives with hollow microstructure have good colloid stability and high cross-linking properties. In order to calculate the relevant parameters more accurately, a 5 kg drop-hammer was chosen for impact sensitivity tests. Results demonstrated these nanoenergetic derivatives samples can be detonated with 12.8 cm. Moreover, the nanoenergetic hollow spherical derivatives are insensitive to spark and friction stimuli. Especially, the friction sensitivity of nanoenergetic derivatives was 0%. The remarkable de-sensitivity of nanoenergetic hollow spherical derivatives provides good evidence for the importance of spherization design and preparation in reducing the sensitivity of energetic materials.

## 4. Conclusions

In summary, we fabricated nanoenergetic hollow spherical HNS derivatives by a one-pot copolymerization strategy. Various characteristics were tested and the results were discussed. A free radical copolymerization reaction process to form HNS derived oligomers was inferred. The unique hollow structure of spherical nanoparticles was verified. The nanoenergetic hollow spherical HNS derivatives with high regularization, uniform size distribution and good monodispersity showed a distinctive thermal decomposition and the good safety performance of the spherization nanotechnology. Although, the reaction involved in this work is very complicated and the mechanism is not yet fully understood, more investigations including theoretical and experimental studies are needed to optimize spherization nanotechnology. There is no doubt that this work introduces a new concept for the design and preparation of spherical energetic materials and adjusts their properties to meet expectations.

## Figures and Tables

**Table 1:** Impact, Spark, and Friction Sensitivity of the sample, raw HNS and other explosives.

Sample	Impact Sensitivity (H_50_/cm)	Friction Sensitivity (%)	Spark Sensitivity (E_50_/J)	
sample	>112.2/12.8 *	0	5.19	
HNS	39.5	28	1.11	
RDX	99.1	50	-	[41]
HMX	63.0	58	-	[41]
CL-20	29.4	88	-	[41]

* Drop weight, 5 kg; Sample mass, 30 mg.
